# Mitochondrial DNA Variation and Selfish Propagation Following Experimental Bottlenecking in Two Distantly Related *Caenorhabditis briggsae* Isolates

**DOI:** 10.3390/genes11010077

**Published:** 2020-01-10

**Authors:** Josiah T. Wagner, Dana K. Howe, Suzanne Estes, Dee R. Denver

**Affiliations:** 1 Cancer Early Detection Advanced Research (CEDAR) Center, Knight Cancer Institute, Oregon Health & Science University, Portland, OR 97201, USA; 2 Department of Integrative Biology, Oregon State University, Corvallis, OR 97331, USA; howeda@science.oregonstate.edu (D.K.H.); denvedee@science.oregonstate.edu (D.R.D.); 3 Department of Biology, Portland State University, Portland, OR 97201, USA; estess@pdx.edu

**Keywords:** mitochondria, selfish DNA, mutation, bottleneck, nematode, heteroplasmy

## Abstract

Understanding mitochondrial DNA (mtDNA) evolution and inheritance has broad implications for animal speciation and human disease models. However, few natural models exist that can simultaneously represent mtDNA transmission bias, mutation, and copy number variation. Certain isolates of the nematode *Caenorhabditis briggsae* harbor large, naturally-occurring mtDNA deletions of several hundred basepairs affecting the *NADH dehydrogenase subunit 5* (*nduo-5*) gene that can be functionally detrimental. These deletion variants can behave as selfish DNA elements under genetic drift conditions, but whether all of these large deletion variants are transmitted in the same preferential manner remains unclear. In addition, the degree to which transgenerational mtDNA evolution profiles are shared between isolates that differ in their propensity to accumulate the *nduo-5* deletion is also unclear. We address these knowledge gaps by experimentally bottlenecking two isolates of *C. briggsae* with different *nduo-5* deletion frequencies for up to 50 generations and performing total DNA sequencing to identify mtDNA variation. We observed multiple mutation profile differences and similarities between *C. briggsae* isolates, a potentially species-specific pattern of copy number dysregulation, and some evidence for genetic hitchhiking in the deletion-bearing isolate. Our results further support *C. briggsae* as a practical model for characterizing naturally-occurring mtgenome variation and contribute to the understanding of how mtgenome variation persists in animal populations and how it presents in mitochondrial disease states.

## 1. Introduction

Animals that rely on the mitochondrial electron transport chain (ETC) for generating the majority of their energy inherently depend upon functional mitochondria. These intracellular organelles are unique because they harbor and replicate their own genomes that encode for several essential ETC subunits and components for intramitochondrial protein synthesis [1]. Direct and indirect measurements of mitochondrial genome (mtgenome) evolution in animals have suggested elevated per generation rates of mtgenome mutation when compared to nuclear DNA (nDNA) [2,3,4,5]. Many heritable mitochondrial DNA (mtDNA) variants are now known to have negative effects on organismal fitness and thus mtDNA variation is an important source of genetic disease in animal populations [6,7].

Like nDNA mutations, mutations in mtDNA can be passed from parent to progeny via packaging into germ cells. However, the vast majority of mtDNA is inherited maternally [8]. Also in contrast to nDNA, multiple copies of the mtgenome are found in each cell with nDNA:mtDNA ratios able to deviate widely between normal and pathological states [9]. Single cells or organisms can harbor mtgenome copies of differing sequence, a phenomenon known as mtDNA heteroplasmy, which may be exacerbated by the accelerated mutation rate of mtDNA. Recent work suggests that mtDNA heteroplasmy is remarkably common and that relative abundances of specific variants can affect physiology, disease, lifespan, and/or distort sex ratios [10,11,12,13]. Importantly, mtDNA variant ratios in offspring do not always recapitulate parental heteroplasmy composition and there is accumulating evidence that not all mtDNA elements have equal probability of entering daughter somatic cells or the germline [14,15,16,17]. Strongly deleterious mtDNA variants passed to offspring would be expected to be rapidly selected against. Yet, evolution can favor transmission of some deleterious mtDNA variants provided they have replication or transmission advantages compared to other mtDNA variants. DNA elements that are preferentially transmitted despite their neutral or negative effect on fitness are more broadly known as “selfish” or “parasitic” DNA [18], and there is increasing evidence that mtDNA can behave as a bona fide selfish DNA element across a diverse array of eukaryotes [14,19,20,21]. Understanding these selfish elements alongside mtDNA mutation spectra is of particular interest from an evolutionary standpoint because the abundance of certain mtDNA variants may cause accelerated mito-nuclear genome incompatibility in populations, in turn driving cyto-nuclear co-evolution and potentiating “Red Queen” dynamics [22,23].

Despite their potential prevalence, examples of naturally occurring selfish mtDNA element transmission are rare because their negative fitness effects make them prone to extinction. Selfish mtDNA elements remain cryptic unless they are associated with an obvious phenotype that warrants investigation, and therefore are understudied within the context of background mtDNA mutation rates. The nematode *Caenorhabditis briggsae* has been developed over the past decade as a model to address these challenges and has practical advantages compared to other animal systems. Often coexisting in nature with its better-known relative *Caenorhabditis elegans*, *C. briggsae* shares several experimentally useful traits with *C. elegans* in that they are easily maintained under laboratory conditions, are amenable to age synchronization, have a rapid life cycle, and primarily reproduce as self-fertilizing hermaphrodites [24,25]. Several natural isolates of *C. briggsae* are known to harbor large heteroplasmic mtDNA deletions of mitochondrial NADH dehydrogenase subunit 5 (*nduo-5*) that eliminate several hundred bases from the 5′ end of the gene [26]. This deletion event (*nad5*Δ) is associated primarily with direct repeat motifs shared between the *nduo-5* gene and an upstream 325-344 bp pseudogene element (Ψnad5-2) that has high homology to *nduo-5* [27]. Importantly, *nad5*Δ is thought to behave as a selfish DNA element that adversely affects fitness through impaired mitochondrial functioning and displays transmission bias in small population sizes expected to promote genetic drift [28,29,30]. Because isolates of *C. briggsae* lacking the Ψnad5-2 mtDNA element do not accumulate *nad5*Δ, this model system provides an opportunity to compare how the spectra of new mtDNA mutations may vary among isolates of the same species.

Although the mutation dynamics of *C. briggsae* mtDNA has been a subject of investigation for over a decade, past studies have primarily relied on PCR fragment analysis and Sanger sequencing. These techniques are effective at distinguishing major base substitution or deletion events in *C. briggsae* isolates but may miss low frequency heteroplasmic events, especially at complex variant sites such as *nad5*Δ. Several studies have identified the potential for isolate-specific or species-specific mutation profiles in nematodes, as well as putative hotspots for mutation, but the model of mtDNA mutation across generations in *C. briggsae* remains incomplete [3,17,31,32]. Previous work by Phillips et al. [29] suggested that although there is the potential for multiple *nad5*Δ variants to form, *C. briggsae* isolates that accumulate *nad5*Δ appear to harbor only a single *nad5*Δ variant at the end of experimental bottlenecking. It is currently unknown if extreme drift conditions promote the accumulation of a single *nad5*Δ variant, or if several *nad5*Δ variants can also remain in the population at residual frequency. In addition, because *nad5*Δ behaves as a selfish DNA element, mtgenomes that harbor the element may also transmit other linked mutations as a single unit; i.e., “genetic hitchhiking” [33], but evidence for these events in *C. briggsae* mtDNA is lacking. Finally, Wernick et al. [31] observed that experimental population bottlenecking significantly increases the mtDNA:nDNA ratio in age-synchronized *C. elegans*. This finding was attributed to the effects of accumulating mitochondrial dysfunction over the bottlenecking period, but it was later shown that *C. elegans* mutants undergoing adaptive evolution in large population sizes can also experience similar increases in relative mtDNA copy number [34]. Whether the elevated mtDNA copy number accompanying experimental evolution observed for *C. elegans* is generalizable to other species remains unknown.

To address these knowledge gaps, we compare the mtDNA mutation profiles of two *C. briggsae* isolates, AF16 (Clade I, tropical) and ED3101 (Clade III, equatorial), following extreme population bottlenecking. AF16 and ED3101 are phylogenetically distant, with AF16 representative of natural isolates containing the Ψnad5-2 pseudogene and heteroplasmy for *nad5*Δ, while ED3101 is representative of those lacking Ψnad5-2 and *nad5*Δ [26,30]. Clade designations and phylogenetic inferences are from Raboin et al. [27] Other wildtype *Caenorhabditis* species including *C. elegans* also lack Ψnad5-2 and *nad5*Δ, and therefore the mtgenome of ED3101 is representative of those throughout the genus [26]. To mitigate the effects of evolutionary forces other than DNA mutation, we generated several mutation-accumulation (MA) lines of AF16 and ED3101 [4]. These MA lines were independently evolved by randomly selecting single hermaphrodite individuals from a progenitor pool to initiate the next generation for up to 50 generations. To determine the mtDNA mutation and mtDNA copy number profiles of age-synchronized MA and progenitor lines, we then applied high-throughput shotgun sequencing on total DNA. Our results suggest several convergent and divergent aspects of mtDNA mutation between AF16 and ED3101 MA, which have implications for the model of mtDNA evolution in *C. briggsae*, and suggest that isolate- and species-specific mtDNA dynamics can potentially drive fitness changes and speciation [35].

## 2. Materials and Methods 

### 2.1. Generation of C. briggsae Mutation Accumulation Lines

A total of 24 MA lines per isolate were initiated from the offspring of a single progenitor hermaphrodite for both AF16 and ED3101 *C. briggsae*, and then passaged through single-nematode bottlenecks as previously described [36]. Each MA line was passaged for a maximum of 50 generations. Lines with fewer than 50 generations were the result of the selection of a nematode that failed to reproduce, causing a replacement nematode to be selected from the previous generation and resulting in the loss of one generational time point.

### 2.2. Total DNA Extraction and Sequencing

For each *C. briggsae* isolate, mixed-staged nematodes were collected from multiple plates of the progenitor and five randomly selected MA lines. Each line was age synchronized (Wood, 1988) and the first larval (L1) stage collected for DNA extraction using the Qiagen DNeasy Blood & Tissue kit (Qiagen #69504, Valencia, CA, USA) as previously described (Wernick et al., 2016). Libraries for each MA line were individually prepared with 1.5 µg of total genomic DNA following Illumina TruSeq protocol (Illumina, Inc., San Diego, CA, USA), then pooled together for two runs—one for each isolate. Sequencing (250 bp paired-end) was performed on an Illumina MiSeq Genome Analyzer at Oregon State University’s Center for Genome Research and Biocomputing. All raw read sequences were deposited into the NCBI Sequence Read Archive under their respective BioProject identifiers (Supplemental Table S1A). 

### 2.3. Raw Read Processing

Read quality and complexity was assessed using FastQC v0.11.3 [37]. Adapters were removed from raw reads using Trimmomatic v0.33 (seed mismatches = 2, palindrome clip threshold = 30, simple clip threshold = 7, min adapter length = 1, keepBothReads = TRUE) [38]. The remainder of read preprocessing and mapping was performed using their respective plugins in Geneious v11.0.5 (Biomatters, Ltd., Auckland, New Zealand). Paired reads were merged using BBMerge v8.81 with default settings [39]. Reads that could not be merged successfully were discarded. For each library, exact duplicate reads were removed using the Dedupe plugin v37.28 (ac = f).

### 2.4. Reference Sequences for C. briggsae AF16 and ED3101 Isolates

Mtgenome reference sequences for *C. briggsae* AF16 (accession AC186293) and ED3101 (accession EU407790) were obtained from NCBI Genbank. Nuclear reference DNA (nDNA) sequences for *C. briggsae* AF16 *ama-1* (chr IV: 15412313-15431314), *efl-2* (chr II: 11371089-11372629), and ego-1 (chr I: 620999-640478) were obtained from the WormBase CB4 assembly GCA_000004555.3 annotation version WS267.

### 2.5. Extension of ED3101 mtGenome Reference 

To improve mapping quality at the AT-rich regions of the ED3101 mtgenome reference, we mapped all ED3101 progenitor reads to the ED3101 mtgenome using Geneious iteratively to the best consensus for 10 times (≤5% mismatch, map quality 30, no gaps, ≤10% gaps per read as a percentage of read length, max gap size = 15, word length = 18). Regions with coverage < 2 standard deviations (SDs) of the mean coverage of the entire mtgenome were excluded from the final reference sequence.

### 2.6. Generation of ED3101 and AF16 Nuclear Genome Reference Regions

All ED3101 progenitor and MA line reads were mapped to the AF16 reference genome assembly CB4 *ama-1*, *efl-2*, and *ego-1* nDNA sequences using Geneious mapper (≤20% mismatch, ≤10% gaps per read as a percentage of read length, max gap size = 15, word length = 18, no multiple best matches). Contigs were assembled from the mapped reads using Geneious de novo assembler with default settings. The longest contig from each ED3101 assembly was aligned pairwise to its respective AF16 gene using MUSCLE v3.8.425 and the longest continuous region with >85% identity between the two isolates was extracted from the aligned sequences [40]. The resulting *ama-1*, *efl-2*, and *ego-1* regions for each isolate were used as the reference loci for coverage normalization.

### 2.7. Read Mapping to mtGenomes and Variant Discovery

For each library, reads were mapped to their respective isolate reference mtgenomes using the BBMap aligner v37.28 plugin in Geneious (k = 8, maxindel = 2000, tipsearch = 2000, slow minratio = 0.8, ambiguous = toss). Alignment bam files were sorted using samtools v1.5 [41]. Reads surrounding the large canonical Ψnad5-2-to-*nduo-5* deletion region in the AF16 MA lines were realigned using GATK v3 IndelRealigner tool [42]. Mtgenome variations were determined using the find variations/SNPs tool in Geneious requiring a minimum coverage of 100, at least 4 raw supporting reads, approximated *p* < 1 × 10^−6^, and a strand-bias *p* value of >10^−5^ when exceeding 65% bias. In a separate analysis, the minimum coverage, raw read, and *p* value requirements were removed to check for mtDNA variants that may have been segregating at extremely low frequency in all lines. The most frequent bases in the progenitor libraries were set to be the reference bases for mapping and variant calling. Putative variant sites were manually inspected for alignment quality. To determine shared variant sites between isolates and MA lines, AF16 and ED3101 mtgenomes were aligned pairwise using MUSCLE v3.8.425. 

### 2.8. Mutation Rate Analysis

MtDNA mutation rates for ED3101 and AF16 MA lines were calculated using the equation:

*μ* = *m*/(Ln*T*), (1)

where *μ* is the base substitution rate or homopolymer indel event rate (per nucleotide site per generation), *m* is the number of observed mutations, *L* is the number of MA lines, *n* is the number of scanned mtDNA nucleotide sites, and *T* is the average number of MA generations for the isolate [43,44]. To estimate *m*, we calculated the difference in mutation frequencies between the MA lines and progenitor at each variant site and summed the absolute value of the differences [32]. For base substitution rates, *n* was the total number of mtgenome bases excluding the AT-rich region, which we did not consider for variant calling. For homopolymer indel events, *n* was determined by summing all A or T homopolymer regions eight bases or greater and excluding the AT-rich region. We included mononucletide runs of only eight bases or greater as previous work suggests that this is the threshold required for replication slippage to occur [31,32,45]. A or T homopolymer stretches of 8 or more bp were identified using seqkit v0.10.1 [46]. As previously described by [3,32], standard errors were estimated by using the equation:

[*μ*/(Ln*T*)]^1/2^. (2)

### 2.9. Determination of Relative mtDNA Copy Number

For each library, the raw coverage of each mtgenome, and *ama-1*, *efl-2*, and *ego-1* nDNA sequence was determined using bbpileup v38 with default settings. The AT-rich intergenic region of the mtgenomes were discarded and raw mtgenome sites were normalized to the average coverage of the *ama-1*, *efl-2*, and *ego-1* reference sequences. Visualization of coverage and statistical comparisons were performed using GraphPad Prism 8.0.2 (GraphPad Software, San Diego, CA, USA). The D’Agostino and Pearson test for normal (Gaussian) distribution was performed using the normalized coverage positions for each sample. Subsequently, the non-parametric Kruskal–Wallis test was followed by Dunn’s multiple comparisons test to compare MA line normalized mtDNA coverages to those of the appropriate progenitor. The Kolmogorov–Smirnov test, which does not assume Gaussian distributions, was used to test for significant difference in normalized mtDNA coverage distributions from AF16 and ED3101 isolates. Statistical significance was determined at *p* < 0.05.

## 3. Results

### 3.1. Progenitor and MA Line Sequencing

Five MA lines for each of the AF16 and ED3101 *C. briggsae* isolates were passaged between 42 and 50 generations (Table 1, Supplemental Table S1A). Some MA lines could not be passaged for the maximum 50 generations due to extinction events during the MA process. Along with one progenitor line for both isolates, we sequenced total DNA using two sequencing runs for each sample. The first sequencing run ended 47 bases early on the reverse strand. However, because the first and second runs had similar FastQC metrics and overall rates of read merging, we combined the first and second runs into a single data set to increase total read depth. After raw read processing, merging, and duplicate read removal, ~2 million–4 million reads were used as mapping input for each sample (Table 1). Raw reads have been made available on the NCBI Sequence Read Archive (Supplemental Table S1A).

### 3.2. Exclusive and Shared mtGenome Variation Sites in ED3101 and AF16 Lines

**Figure 1:**
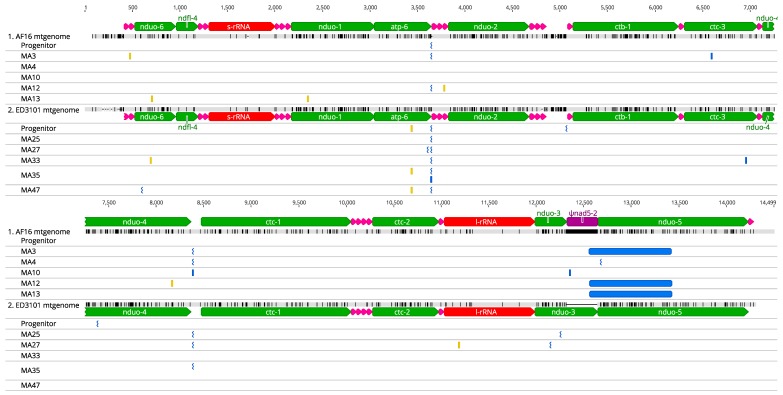
Mitochondrial DNA heteroplasmy in AF16 and ED3101 *C. briggsae* MA lines. The AT-rich region is omitted in alignment and position numbers are relative to the beginning of the alignment. For heteroplasmy events in the progenitor and MA lines, SNPs are represented in orange while insertion (jagged symbol) or deletion (straight symbol) events are represented in blue. Alignment consensus disagreements between AF16 and ED3101 reference mitochondrial genome sequences are in black, while agreements are in grey (overall 93.5% pairwise sequence identity).

We were able to unambiguously extend the ED3101 reference sequence AT-rich region 402 bases upstream to tRNA-Pro (ED3101 average coverage = 172, coverage range = 40–256) using the iterative mapping approach. However, we were unable to extend the AT-rich region downstream of the ED3101 *nduo-5*. Overall, we observed average raw mtgenome coverage between 200×–361× between all AF16 and ED3101 progenitor and MA lines (Table 1). The most frequent AF16 and ED3101 progenitor mtDNA sequences did not differ from their respective mtDNA references taken from NCBI. Across all isolates and lines (both progenitor and MA lines), variant analysis revealed 39 variant sites that met our criteria (Figure 1; Table 2 and Table 3). Total variants for each of the progenitor and MA lines are summarized in Figure 3. Of these 39 sites, 17 variants occurred in coding regions resulting in nonsynonymous mutations, 38 were heteroplasmic, and one was homoplasmic. The majority (26/39) of the variant sites occurred as indel events in homopolymer regions with at least five adenosine or thymine bases, and occurred between frequencies of 0.013 and 0.278. Finally, 11/39 variants were not found in that MA line’s progenitor, or any other MA line from the same isolate, at any frequency, suggesting that they had arisen de novo during laboratory evolution (Table 2; Supplemental Tables S1B and S1C).

Using MUSCLE aligner, AF16 and ED3101 mtgenomes aligned with 93.5% pairwise identity (Figure 1). We observed two locations at which homopolymer indel variation sites were shared between multiple samples from the AF16 and ED3101 isolates. The first occurred at aligned position 3643 (original AF16 position 3567; original ED3101 position 3618) at the 3′ end of the AF16 and ED3101 *atp-6* coding region, resulting in a loss or gain of an adenosine residue at frequencies between 0.013–0.266. Half of the AF16 lines (progenitor, MA3, and MA12) and all six ED3101 lines (progenitor and the five MA lines) shared this insertion variant; one ED3101 line (MA35) also had a deletion variant at this site (Table 2). It should be noted that the AF16 MA10 and MA13 lines had overall lowest mtDNA coverage and therefore the inability to detect the progenitor heteroplasmy at this site may reflect insufficient depth rather than complete loss of the variant. However, these *atp-6* 3′ indel events are predicted to have no effect on the resulting *atp-6* protein because they occur after the TAA stop codon sequence. The second shared homopolymer indel variation site occurred at aligned position 8384 (original AF16 position 8305; original ED3101 position 8357) at frequencies between 0.014 and 0.278. At this site, two out of six AF16 lines (MA3 and MA4) shared this insertion variant, one AF16 line (MA10) had a deletion variant, and three out of six ED3101 lines (MA25, MA27, and MA35) had this insertion variant. For both isolates, aligned position 8384 occurs in an intergenic region between *nduo-5* and *ctc-1* (cytochrome c oxidase subunit I) and is predicted to have no functional effect. In total, we observed five deletion and 21 insertion variants at these homopolymer sites.

Although less frequent than the homopolymer indel variants, we also observed several SNPs in the AF16 and ED3101 lines (Table 2). Nine of the ten mtDNA SNP sites discovered within the AF16 and ED3101 MA or progenitor lines were determined to be G → T transversions or C → T transitions. For the AF16 isolate, we observed five SNPs in total among the MA3, MA12, and MA13 lines that were exclusive to each line and not present in the progenitor at significant frequency. Two of these five AF16 MA line SNPs were predicted to have protein coding effects (*nduo-4* or *nduo-1*), one was silent (*nduo-6*), and two were predicted to occur in tRNAs (tRNA-Val and tRNA-Ser). For the ED3101 isolate, we observed five SNPs in total among the progenitor, MA27, MA33, MA35 and MA47 lines. At ED3101 SNP site position 3414, occurring within a second codon base position of mitochondrially-encoded ATP Synthase Membrane Subunit 6 (*atp-6*), the progenitor line was determined to have 60% cytosine (TCA, serine) and 40% thymine (TTA, leucine). Three of the ED3101 lines, MA25, MA27, and MA33 fixed for cytosine at this site (TCA, serine), while MA47 fixed for thymine at this site (TTA, leucine) and MA35 remained heteroplasmic (70% cytosine, 30% thymine). Two other SNPs were discovered in ED3101 MA27 or MA33, with the first occurring in the large rRNA subunit, and the other predicted to have a protein coding effect in *nduo-6*, respectively.

**Figure 2:**
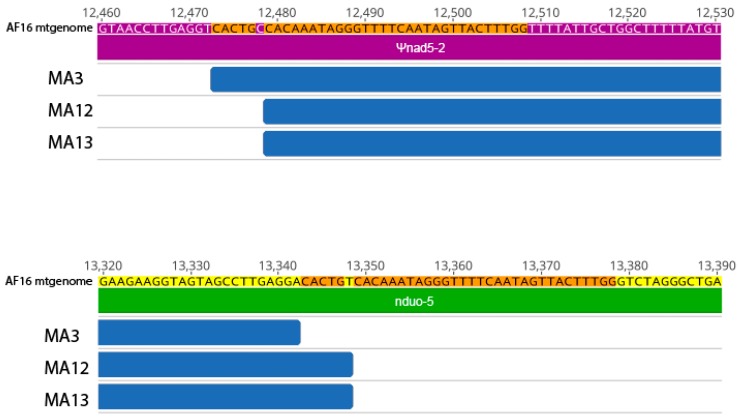
Large deletions harbored by AF16 MA3, MA12, and MA13 mitochondrial genomes. Annotations and base positions are based on the reference AF16 mtgenome. The top region shows positions 12,460–12,530, which are within ψnad5-2, while the bottom region shows positions 13,320–13,390, within *nduo-5*. The deletion regions, referred to collectively as *nad5*Δ and represented in blue, are 870 bp in length and span from ψnad5-2 into *nduo-5*. While MA13 and MA12 share the same *nad5*Δ, the deletion in MA3 is shifted +6 bases. None of the AF16 MA lines had evidence for more than one *nad5*Δ deletion variant. The 5 bp and 30 bp direct repeat motifs flanking the deletions are shown in orange.

**Figure 3:**
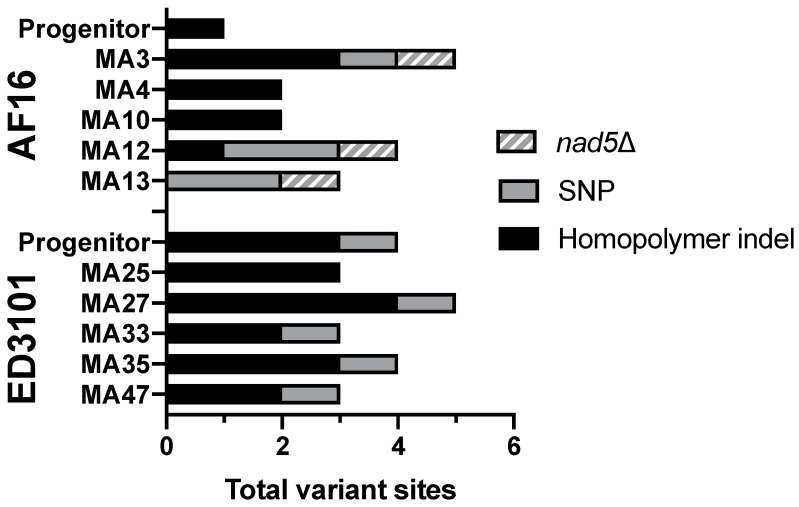
Total number of variant sites for each AF16 and ED3101 MA line and progenitor. The lined bars designate MA lines that also harbored an 870 bp deletion occurring between ψnad5-2 and *nduo-5*.

We did not observe deletions larger than 1 bp in the ED3101 progenitor or any of its MA lines. However, for three of the five AF16 MA lines, we observed an 870 bp mtDNA deletion occurring between ψnad5-2 and *nduo-5* that was also identified subsequently by variant calling on the split reads (Figure 2, Supplemental Figure S1, Table S3). This deletion was found in MA3, MA12, and MA13 at frequencies between 0.651 and 0.918. Conversely, we did not observe evidence of this or any other 870 bp ψnad5-2-to-*nduo-5* deletion in the AF16 progenitor or the other two AF16 MA lines. While this deletion was similar in overall size to the ‘canonical’ AF16 ψnad5-2-to-*nduo-5* deletion reported by Howe and Denver (2008), which eliminates the first 786 bp of the 5’ end of *nduo-5*, it eliminated only the first 775 (MA12 and MA13) or 781 (MA3) bp of *nduo-5.* The three aforementioned MA lines also had the highest number of total variant sites among the six AF16 lines (Figure 3). Although the total length of the deletion was the same for the three MA lines, the deletion in MA3 was +6 bases from the start position relative to the first deleted position with MA12 and MA13. A 36 bp direct repeat region was observed at the beginning (position 12,473–12,508) and end (position 13,343–13,378) of the deletion regions. However, a single C/T mismatch at the sixth base of the direct repeat splits the repeat sequence into two smaller (5 bp and 30 bp) exact direct repeat sequences. 

### 3.3. AF16 and ED3101 Share Similar mtDNA Mutation Rates

**Figure 4:**
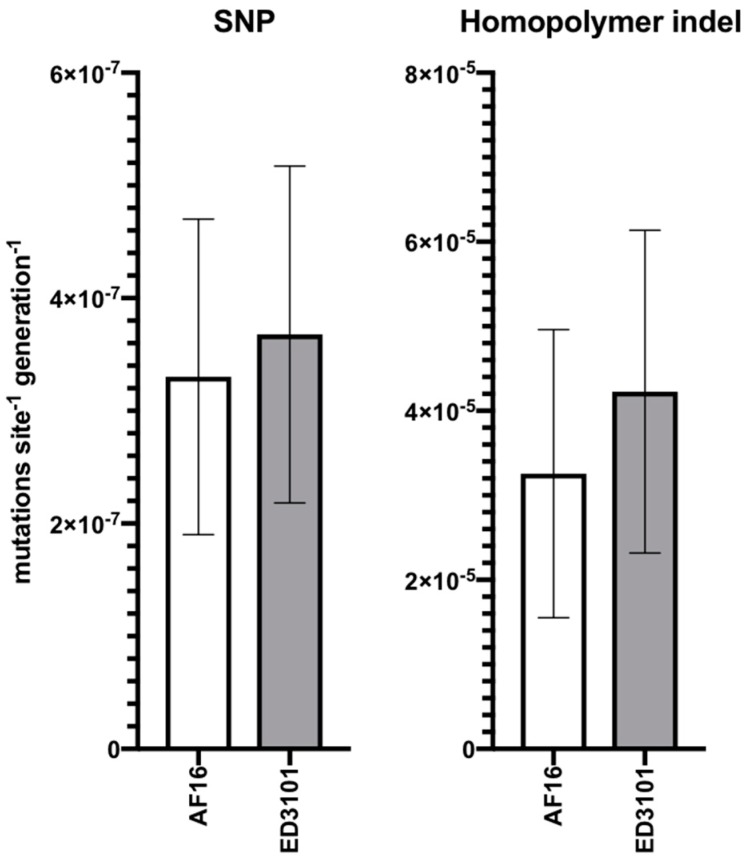
Mutation rate estimates for AF16 and ED3101 MA lines. For each isolate, mutation rates represent the combined estimate that includes their five respective MA lines. Homopolymer indel mutation rates were calculated based on only homopolymer indel variants that were found in runs ≥ 8 bp. Error bars are ± 1 standard error of the mean.

The rate of occurrence of new single base pair mutations estimated for AF16 MA lines was 3.301 (SE = 1.4) × 10^−7^ site^−1^ generation^−1^ while that for ED3101 MA lines was 3.678 (SE = 1.495) × 10^−7^ site^−1^ generation^−1^ (Figure 4, left). The homopolymer indel rate at mononucleotide runs of eight bases or more was estimated at 3.256 (SE = 1.703) × 10^−5^ site^−1^ generation^−1^ for AF16 MA lines and 4.228 (SE = 1.91) × 10^−5^ site^−1^ generation^−1^ for ED3101 MA lines (Figure 4, right). Mutation rate estimates generated from each isolate had overlapping SE intervals. The number of A/T bases contained within mononucleotide runs of eight or more, outside of the AT-rich region, was 92 and 95 bases for AF16 and ED3101, respectively.

### 3.4. Relative Mitochondrial Genome Copy Number Decreases Following MA

**Figure 5:**
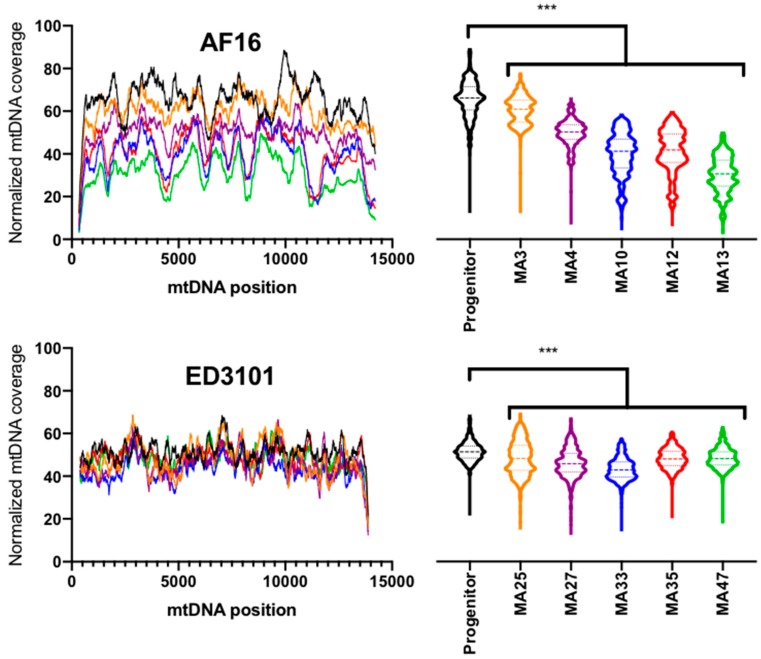
Relative mtDNA coverage in AF16 and ED3101 *C. briggsae* MA lines. MtDNA content is reduced relative to progenitors in AF16 (top panels) and ED3101 (bottom panels) lines following MA. The AT-rich region of the mitochondrial genomes was omitted from analysis. Colors for the mtDNA position coverage plots (left panels) correspond to their respective progenitor and MA line colors for the violin plots (right panels). MA lines were compared to progenitor lines using Kruskal–Wallis test followed by Dunn’s multiple comparisons test (***, *p* < 0.001).

Using the CB4 reference assembly, we assembled nDNA sequence regions for ED3101 *ama-1*, *efl-2*, and *ego-1* with 92V 99% pairwise similarity to AF16 (Supplemental Figure S2, Supplemental Table S1D). Overall, we found that the average mtDNA coverage following normalization was between 30.3 and 65.4 for the samples used in this study (Table 1; Figure 5, left). All five AF16 MA lines were determined to have significantly different normalized mtDNA coverage than the progenitor, with AF16 MA lines having between 5.2 and 35.1 less average coverage compared to the progenitor (Kruskal–Wallis test followed by Dunn’s multiple comparisons test, *p* < 0.0001, Figure 5, top right). AF16 MA10 and MA13 had the lowest overall coverage of both isolates and MA lines. Although to a lesser extent than AF16, all ED3101 MA lines were determined to have significantly lower (3.07–8.29) normalized mtDNA coverage than the progenitor (Kruskal–Wallis test followed by Dunn’s multiple comparisons test, *p* < 0.0001, Figure 5, bottom right). Notably, while normalized mean coverage for AF16 (46.24×) and ED3101 (46.91×) were similar, the standard deviation for the AF16 lines (SD = 16.64×) was nearly double that of the ED3101 lines (SD = 7.69×). The coverage of the AF16 and ED3101 normalized mtgenomes was determined to have significantly unequal distributions (Kolmogorov–Smirnov test, *p* < 0.0001).

## 4. Discussion

The assumption of taxon-independent mtDNA mutation patterns across generations has long been used as justification for using mtDNA in species barcoding. However, this assumption remains controversial [47]. Our work agrees with accumulating evidence that, while certain aspects of mtDNA mutation may be conserved, species- and isolate-specific mutation profiles and mtgenome copy number dynamics can occur. In the present study, we were able to reliably identify mtDNA variants within MA lines from two *C. briggsae* isolates (Table 1) occurring at frequencies as low as 1.4% (Table 2, Figure 1). Our mtDNA SNP results for AF16 and ED3101 lines agree with the overall extreme mutation bias towards A and T mutations in *Drosophila melanogaster* [5] and *C. elegans* mtDNA [3,48]. It is tempting to attribute the observed mutational bias towards A and T bases for both *C. briggsae* isolates to an increased abundance of unrepaired 8-oxoguanine and deaminated cytosine bases [49], but the low sample size precludes meaningful analysis of mutational bias mechanisms. 

Using the MA approach, we were able to observe the fate of mtDNA heteroplasmy in the *C. briggsae* isolates following up to 50 generations of evolution. There are three possible fates for a heteroplasmic SNP in an evolving population: the SNP fixes for wildtype, the SNP fixes for a variant, or the SNP remains heteroplasmic. Consistent with this the frequencies of this SNP being shaped by random genetic drift, as expected under mutation-accumulation experimental conditions, we observed all three potential outcomes at position 3414 for ED3101. Three ED3101 lines (MA25, MA27, and MA33) fixed for the reference, one (MA47) fixed for the variant, and one (MA35) remained heteroplasmic at nearly the same level as the progenitor. Previous work by Howe et al. [32] using two other *C. briggsae* isolates, PB800 and HK104 (Clade II, Temporal), identified a trend for SNPs found to be heteroplasmic within *C. briggsae* progenitors to remain heteroplasmic after 250 generations of MA. This result contrasted with studies of wildtype *C. elegans*, which found only fixed differences in 214-generation MA lines [3], and heteroplasmy in only one out of five 250-generation MA lines [31]. Our results suggest that some mtDNA SNPs can be rapidly fixed (<50 MA generations) within a particular Clade I *C. briggsae* isolate, ED3101, potentially providing an example of an intermediate level of mtDNA bottlenecking between *C. elegans* and Clade II *C. briggsae* isolates.

In addition to the five SNPs we observed in the AF16 MA lines, we also identified three MA lines containing an 870 bp deletion (*nad5*Δ) spanning part of ψnad5-2 and the 5′ coding region of *nduo-5* (Figure 2, Supplemental Figure S1, Table 3). Two of these deletion regions had identical boundaries (MA12 and MA13). Our finding agrees with previous work suggesting that the formation of *nad5*Δ in Clade I and Clade II isolates is likely to result from illegitimate recombination between flanking direct repeats, similar to the case with the common large-scale deletions found in human mtDNA [26,50]. Levels of *nad5*Δ reached 65%–90% heteroplasmy in the three MA lines (MA3, MA12, and MA13), passing the predicted threshold of 50% *nad5*Δ that has been proposed to result in significantly higher ROS and lower reproductive fitness [51]. Each of the three lines that carried *nad5*Δ was only observed to have a single dominant form, which has been previously observed in *C. briggsae* MA lines [29,30,32] but yet to be to validated at high-depth single-nucleotide resolution. We were unable to detect *nad5*Δ within the AF16 progenitor, similar to the case with the inbred *C. briggsae* PB800 progenitor used by Howe et al. [32]. There is the possibility that the observed *nad5*Δ variants represent de novo deletion events that independently arose and rapidly accumulated within three of the five (MA3, MA12, MA13) lines. While it is possible that the progenitor may have harbored extremely low levels (<4 raw reads out of >2 million reads) of multiple deletion variants below our detection limit that either propagated or were lost, that we were unable to observe the MA3, MA12, and MA13 *nad5*Δ variants at any frequency for the AF16 progenitor or other MA lines is consistent with these variants having arisen de novo during MA (Supplemental Table S1B). Interestingly, we only observed the presence of SNPs in the three AF16 MA lines that also harbored *nad5*Δ and none in the two lines lacking *nad5*Δ (Figure 3). While the pathogenic effect of the tRNA SNPs is difficult to determine, two of the three coding region SNPs were predicted to have an effect on the resulting protein (Table 2). We propose that the AF16 SNPs of MA3, MA12, and MA13 may have avoided purifying selection by their shared association with *nad5*Δ. However, the short read lengths used in this study limit our ability to confirm if the *nad5*Δ elements are on the same mtgenomes as SNP-bearing ones. Agreeing with previous estimates in *C. briggsae* [32], the overall rate of occurrence of new SNPs in AF16 was not significantly different than that estimated for ED3101 (Figure 4); it therefore seems unlikely that isolate-specific mutation rates affected the MA SNP distributions we observed here. Examples of naturally occurring mtDNA genetic hitchhiking that could potentially accelerate any mitonuclear incompatibility important for species divergence are rare [22,35], but future use of long reads that can span the entire mtgenome could be applied to further examine this potential phenomenon in *C. briggsae*.

Relative to SNPs and *nad5*Δ, the most common polymorphisms observed in both AF16 and ED3101 isolates were indel homopolymer events occurring at A or T sites (Figure 1, Table 2). We estimated the within-isolate rate of homopolymer indel events to be much higher than those for SNPs (>2 SE from mean) but recognize that inherent differences in homopolymer compositions make between-isolate comparisons difficult (Figure 4). Because previous work in *C. elegans* and *C. briggsae* identified mononcleotide runs of eight or more to be hot spots for evolution, a pattern attributed to an increased frequency of template slippage during replication at this threshold, we limited our homopolymer indel mutation analysis to these regions [31,32,45]. However, we observed the majority (21/26) of total homopolymer indel events to occur in mononucleotide runs of less than eight nucleotides; whether these short homopolymer indel events are generated by the same mechanism as those of eight nucleotides or more is unclear. Our overall results support a model wherein many such homopolymer regions are subject to length fluctuations across generations and where some may constitute conserved mutational hot-spots between isolates. Because we removed PCR duplicates prior to mapping, each supporting read is presumed to have been derived from an independent mtDNA molecule and therefore unlikely to be derived from amplified polymerase errors during library prep. It is unclear why evolutionary rates vary across homopolymer regions of similar size. In some instances, we observed homopolymer heteroplasmy in mtDNA coding regions that had higher variant frequency than some non-coding homopolymer regions, even within the same MA line (e.g., ED3101 MA25, Table 2). Our data suggest that homopolymer runs of similar size may have equal potential for evolution in *C. briggsae*, although this hypothesis needs to be tested by sampling lines at intermediate generational time points as they progress through MA.

The high-throughput sequencing strategy used here allows for single-nucleotide resolution of mtDNA variants but also allows us to estimate the fraction of DNA derived from mitochondria versus nDNA in the same population. We limited the effects of nematode developmental stage on mtDNA copy number by using only age-synchronized L1 animals [52]. Using L1-stage animals, Wernick et al. [31] and [34] reported significant increases in mtDNA:nDNA ratios following MA in both wildtype *C. elegans* and a *C. elegans* mutant with an ETC Complex I deficiency. Although knowledge of the association between increased mtDNA copy number and oxidative stress is limited mostly to human tissues and subjects [53,54], this finding was suggested to be due to increasing mitochondrial dysfunction or ROS associated with MA that may drive increasing copy numbers as compensation. The present study is the first to demonstrate mtDNA copy number dynamics following MA in *C. briggsae* and we found that MA results in significantly lower mtDNA:nDNA ratios in both AF16 and ED3101 isolates, in contrast to the findings in *C. elegans* (Figure 5). While the reduction in relative mtDNA copy number was most severe in several AF16 MA lines, the degree of reduction did not appear to be driven by whether or not the MA lines were heteroplasmic for *nad5*Δ (Figure 3). Unexpectedly, we observed a higher degree of mtgenome coverage variation across the AF16 progenitor and MA lines compared to ED3101 lines (Table 1). This uneven sequencing coverage across the AF16 mtgenome may have been caused by an increased presence of linear mtDNA fragments generated by mtDNA degradation and/or incomplete replication [55]. However, we were not able to observe consistent stretches of increased or decreased coverage in the AF16 lines that would provide an obvious mechanism for why the coverage distributions significantly differ from ED3101. Recent work using a mouse model suggests that the absolute number of mtDNA copy, rather than degree of heteroplasmy alone, is an underappreciated and important factor when determining whether or not a mtDNA variant will present with a phenotype [56]. In addition, a recent investigation of selfish mtDNA dynamics in a *C. elegans* strain suggests that maintaining wildtype copy number homeostasis consequently leads to “runaway” levels of the selfish mtDNA element [21]. Perhaps during *C. briggsae* MA there is an overall depression of mtDNA to suppress the total numbers of potentially pathogenic mtDNA variants. Because the *C. briggsae* MA lines were only followed for up to 50 generations, it is currently unknown if subsequent *C. briggsae* MA generations would eventually cause an increase in mtDNA copy number like in *C. elegans*. Whether the difference in mtDNA copy number dynamics between *C. briggsae* and *C. elegans* observed here represents a true species divergence remains to be determined, but it may provide insight into how controlling absolute mtDNA copy number can reduce harmful mtDNA variants and thus potentially have a role in contesting Muller’s Ratchet [57].

## 5. Conclusions

In summary, we characterized low- to high-frequency mtDNA heteroplasmies in two bottlenecked *C. briggsae* isolates. Overall, mtDNA mutation rates between the two isolates were not significantly different. We found that some mtDNA homopolymer indel mutations were prone to evolution across both isolates. In several of the AF16 MA lines, we found evidence for recurring large mtDNA deletions (*nad5*Δ) that have been previously described as selfish DNA elements, but only one deletion boundary appeared to dominate if they occurred. Longer reads will be necessary to further determine if these deletion-bearing mtgenomes tend to carry other polymorphisms with them following bottlenecking. Interestingly, both isolates tended to have reduced normalized mtDNA content following MA, but the mechanism behind this phenomenon remains unclear. Further characterization of mtDNA mutation and mtDNA copy number dynamics in other *C. briggsae* isolates will be important for understanding the behavior of pathological mtDNA inheritance and mtDNA evolution under a variety of population genetic conditions.

## Figures and Tables

**Table 1:** *C. briggsae* isolates and MA lines used for Illumina DNA sequencing.

Isolate	MA	Gen ^a^	Raw Read Pairs ^b^	Input Reads for Mapping ^c^	Raw mtDNA cov ^d^	Norm mtDNA cov ^d^	ama-1 cov ^d^	efl-2 cov ^d^	ego-1 cov ^d^
AF16									
	Progenitor	0	2,603,066	2,366,924	361 (49.7)	65.43 (9.008)	4.4 (3.03)	5.6 (2.86)	6.50 (3.01)
	MA4	49	2,608,998	2,376,290	354.6 (48.55)	49.63 (6.794)	6.0 (2.9)	7.8 (2.62)	7.58 (2.85)
	MA3	46	2,276,257	2,049,378	346.1 (41.63)	60.23 (7.244)	5.5 (3.34)	6.4 (1.36)	5.33 (2.46)
	MA13	50	2,265,875	2,067,734	200.2 (55.59)	30.31 (8.42)	5.9 (2.14)	7.2 (1.11)	6.78 (3.02)
	MA12	42	2,611,375	2,404,412	274.1 (67.58)	41.19 (10.16)	5.2 (2.2)	8.2 (2.73)	6.58 (2.7)
	MA10	48	2,742,185	2,505,013	275.1 (69.28)	39.82 (10.03)	7.8 (2.76)	7.1 (2.57)	5.87 (2.35)
ED3101									
	Progenitor	0	3,503,305	3,217,360	299.8 (27.94)	51.46 (4.797)	5.8 (3.36)	6.2 (2.32)	5.45 (2.37)
	MA25	50	3,915,576	2,969,457	286 (47.68)	48.39 (8.068)	5.5 (3.39)	7.1 (2.66)	5.13 (2.72)
	MA27	47	4,010,572	3,430,620	302.1 (45.25)	46.06 (6.901)	6.8 (3.25)	6.3 (2.44)	6.50 (3.63)
	MA33	50	4,104,196	3,245,243	264.4 (33.73)	43.17 (5.507)	5.8 (2.98)	6.7 (2.39)	5.80 (3.17)
	MA35	50	3,573,465	3,250,232	279.2 (28.26)	48.17 (4.876)	6.3 (3.31)	5.9 (1.59)	5.18 (2.03)
	MA47	47	3,699,368	3,393,866	271 (27.61)	48.3 (4.92)	6.0 (3.29)	5.4 (3.05)	5.43 (2.74)

^a^ Number of generations MA line was passaged. ^b^ Total number of raw reads following the combination of both sequencing runs. ^c^ Total number of reads following trimming and merging that were used as input for mapping to mtDNA and genomic region. ^d^ For mtDNA coverage, the AT-rich region was omitted from analysis. To calculate normalized mtDNA coverage, the raw mtDNA coverage was divided by the average coverage of *ama-1*, *efl-2*, and *ego-1*. Average coverage metrics are followed by SD in parenthesis.

**Table 2:** Identified homopolymer indels and SNPs in AF16 and ED3101 *C. briggsae* MA lines.

**Homopolymer Indels**									
**Isolate**	**MA**	**Position ^a^**	**Change ^b^**	**Cov ^c^**	**Variant Frequency**	***p*-value**	**Variant Raw Count**	**Gene or Feature**	**Protein Effect ^d^**
AF16	Progenitor	3567	(A)10 → (A)11	402	0.015	3.30 × 10^−10^	6	*atp-6*	None
	MA3	3567	(A)10 → (A)11	374	0.013	5.50 × 10^−8^	5	*atp-6*	None
		6515	(T)7 → (T)6 *	289	0.266	2.70 × 10^−69^	77	*ctc-3*	Frame Shift
		8305	(T)9 → (T)10	362	0.014	4.80 × 10^−9^	5	-	-
	MA4	8305	(T)9 → (T)10	341	0.018	3.20 × 10^−11^	6	-	-
		12,601	(T)8 → (T)9 *	333	0.027	7.50 × 10^−18^	9	*nduo-5*	Frame Shift
	MA10	8305	(T)9 → (T)8	196	0.276	5.60 × 10^−50^	54	Ψnad5-2	-
		12,276	(T)5 → (T)4	214	0.084	1.40 × 10^−8^	18	-	-
	MA12	3567	(A)10 → (A)11	340	0.032	2.30 × 10^−22^	11	*atp-6*	None
ED3101	Progenitor	3618	(A)12 → (A)13	302	0.060	1.60 × 10^−40^	18	*atp-6*	None
		5041	(T)9 → (T)10	280	0.061	1.70 × 10^−38^	17	-	-
	MA25	3618	(A)12 → (A)13	216	0.032	9.60 × 10^−15^	7	*atp-6*	None
		8357	(T)9 → (T)10	266	0.026	4.20 × 10^−14^	7	*-*	-
		12,230	(T)9 → (T)10	228	0.057	2.00 × 10^−29^	13	*nduo-3*	Frame Shift
	MA27	3582	(T)6 → (T)7 *	234	0.030	1.70 × 10^−14^	7	*atp-6*	Frame Shift
		3618	(A)12 → (A)13	214	0.051	1.30 × 10^−24^	11	*atp-6*	None
		8357	(T)9 → (T)10	284	0.018	1.40 × 10^−9^	5	*-*	-
		12,124	(T)7 → (T)8 *	238	0.050	1.30 × 10^−26^	12	*nduo-3*	Frame Shift
	MA33	3618	(A)12 → (A)13	233	0.056	2.60 × 10^−29^	13	*atp-6*	None
		6930	(A)6 → (A)5 *	302	0.066	1.20 × 10^−7^	20	*ctc-3*	Frame Shift
		7355	(T)8 → (T)9 *	309	0.019	1.80 × 10^−11^	6	*nduo-4*	Frame Shift
	MA35	3618	(A)12 → (A)11	294	0.112	4.10 × 10^−21^	33	*atp-6*	None
		3618	(A)12 → (A)13	292	0.055	1.30 × 10^−35^	16	*atp-6*	None
		8357	(T)9 → (T)10	287	0.014	6.80 × 10^−8^	4	*-*	-
	MA47	576	(T)8 → (T)9	255	0.278	2.70 × 10^−206^	71	*nduo-6*	Frame Shift
		3618	(A)12 → (A)13	258	0.089	1.60 × 10^−55^	23	*atp-6*	None
**SNPs**									
**Isolate**	**MA**	**Position ^a^**	**Change ^b^**	**Cov ^c^**	**Variant Frequency**	***p*-value**	**Variant Raw Count**	**Gene or Feature**	**Protein Effect ^d^**
AF16	MA3	402	G → T *	143	0.699	0	100	tRNA-Val	-
	MA12	3707	G → T *	337	0.015	1.10 × 10^−8^	5	tRNA-Ser	-
		8092	G → T	217	0.041	1.20 × 10^−18^	9	*nduo-4*	S → I Sub
	MA13	630	C → T	163	0.245	2.00 × 10^−110^	40	*nduo-6*	None
		2270	T → A *	222	0.072	5.80 × 10^−36^	16	*nduo-1*	Truncation
ED3101	Progenitor	3414	C → T	287	0.404	0	116	*atp-6*	S → L Sub
	MA27	11,162	C → T *	291	0.038	3.20 × 10^−24^	11	l-rRNA	-
	MA33	670	G → T *	251	0.068	4.90 × 10^−41^	17	*nduo-6*	S → I Sub
	MA35	3414	C → T	281	0.299	3.50 × 10^−255^	84	*atp-6*	S → L Sub
	MA47	3414	C → T	260	1.000	0	260	*atp-6*	S → L Sub

^a^ Position on reference genome. ^b^ For homopolymer indels, the base is noted in parenthesis with the length of the homopolymer region immediately following. For both homopolymer indels and SNPs, variants found in the MA that do not occur in the progenitor line at any frequency are noted with *. ^c^ Raw coverage. ^d^ Sub, substitution.

**Table 3:** Identified deletion events in AF16 MA lines.

MA	Position ^a^	Change	Cov ^b^	Variant Frequency	*p*-Value	Variant Raw Count	Gene or Feature	Protein Effect
MA3	12,473	-N(870)	261–299	0.651–0.739	6.20 × 10^−291^	209	Ψnad5-2 ..*nduo-5*	Alternative Start Codon, Truncation
MA12	12,479	-N(870)	233–260	0.823–0.918	0	214	Ψnad5-2 ..*nduo-5*	Alternative Start Codon, Truncation
MA13	12,479	-N(870)	167–188	0.793–0.892	1.20 × 10^−228^	149	Ψnad5-2 ..*nduo-5*	Alternative Start Codon, Truncation

^a^ Position on reference genome. ^b^ Raw coverage.

## Supplementary Materials

The following are available online at https://www.mdpi.com/2073-4425/11/1/77/s1, Figure S1: Representative alignment of AF16 MA13 demonstrating split-read mapping across the deleted ψnad5-2 and *nduo-5* region of the AF16 reference mtgenome, Figure S2: Conserved nDNA regions used for normalization in AF16 and ED3101 lines, Table S1: Excel spreadsheet containing raw read statistics, raw read counts for all mtDNA positions containing an identified significant polymorphism found in at least 1 line, and pairwise similarity of nDNA sequence regions used for mtDNA normalization.
