# Supplementary material for: Mitochondrial DNA Variation and Selfish Propagation Following Experimental Bottlenecking in Two Distantly Related Caenorhabditis briggsae Isolates
Source: Genes (Basel). 2020 Jan 10;11(1):77. doi: 10.3390/genes11010077 (PMC7016712; doi:10.3390/genes11010077)
Supplement: Supplementary file 1 [file genes-11-00077-s001.zip › Supplemental Figures.docx]

**Supplemental Figure 1.** Representative alignment of AF16 MA13 demonstrating split-read mapping across the deleted ψ*nad5*-2 and *nduo-5* region of the AF16 reference mtgenome (original reference positions 12,479 – 13,347).

efl-2

ego-1

ama-1

**Supplemental figure 2. Conserved nDNA regions used for normalization in AF16 and ED3101 lines.** Annotated AF16 efl-2, ego-1, and ama-1 genes were obtained from the *C. briggsae* CB4 assembly. ED3101 reads were mapped to the AF16 genes and subsequently reassembled using Geneious. Fragments from the two strains were aligned using MUSCLE. Disagreements between the two aligned sequences are represented in black while agreements are in grey.
